# New Developmental Evidence Clarifies the Evolution of Wrist Bones in the Dinosaur–Bird Transition

**DOI:** 10.1371/journal.pbio.1001957

**Published:** 2014-09-30

**Authors:** João Francisco Botelho, Luis Ossa-Fuentes, Sergio Soto-Acuña, Daniel Smith-Paredes, Daniel Nuñez-León, Miguel Salinas-Saavedra, Macarena Ruiz-Flores, Alexander O. Vargas

**Affiliations:** Laboratorio de Ontogenia y Filogenia, Departamento de Biología, Facultad de Ciencias, Universidad de Chile, Santiago, Chile; Harvard Medical School, United States of America

## Abstract

From early dinosaurs with as many as nine wrist bones, modern birds evolved to develop only four ossifications. Their identity is uncertain, with different labels used in palaeontology and developmental biology. We examined embryos of several species and studied chicken embryos in detail through a new technique allowing whole-mount immunofluorescence of the embryonic cartilaginous skeleton. Beyond previous controversy, we establish that the proximal–anterior ossification develops from a composite radiale+intermedium cartilage, consistent with fusion of radiale and intermedium observed in some theropod dinosaurs. Despite previous claims that the development of the distal–anterior ossification does not support the dinosaur–bird link, we found its embryonic precursor shows two distinct regions of both collagen type II and collagen type IX expression, resembling the composite semilunate bone of bird-like dinosaurs (distal carpal 1+distal carpal 2). The distal–posterior ossification develops from a cartilage referred to as “element x,” but its position corresponds to distal carpal 3. The proximal–posterior ossification is perhaps most controversial: It is labelled as the ulnare in palaeontology, but we confirm the embryonic ulnare is lost during development. Re-examination of the fossil evidence reveals the ulnare was actually absent in bird-like dinosaurs. We confirm the proximal–posterior bone is a pisiform in terms of embryonic position and its development as a sesamoid associated to a tendon. However, the pisiform is absent in bird-like dinosaurs, which are known from several articulated specimens. The combined data provide compelling evidence of a remarkable evolutionary reversal: A large, ossified pisiform re-evolved in the lineage leading to birds, after a period in which it was either absent, nonossified, or very small, consistently escaping fossil preservation. The bird wrist provides a modern example of how developmental and paleontological data illuminate each other. Based on all available data, we introduce a new nomenclature for bird wrist ossifications.

## Abstract 2

A new study that integrates developmental and paleontological data reveals previously unsuspected evolutionary transformations during the emergence of the bird wrist, consistent with its derivation from non-avian dinosaurs.

## Author Summary

When birds diverged from nonavian dinosaurs, one of the key adaptations for flight involved a remodelling of the bones of the wrist. However, the correspondence between bird and dinosaur wrist bones is controversial. To identify the bones in the bird wrist, data can be drawn from two radically different sources: (1) embryology and (2) the fossil record of the dinosaur–bird transition. Currently, identifications are uncertain, but new developmental data can help resolve apparent conflicts. The modern bird wrist comprises four ossifications, arranged roughly in a square with its sides running proximal/distal and anterior/posterior. Our study integrates developmental and paleontological data and clarifies the relationship between each of these four ossifications and those found in nonavian dinosaurs. This integrative approach resolves previous disparities that have challenged the support for the dinosaur–bird link and reveals previously undetected processes, including loss, fusion, and in one case, re-evolution of a transiently lost bone.

## Introduction

**Figure 1:**
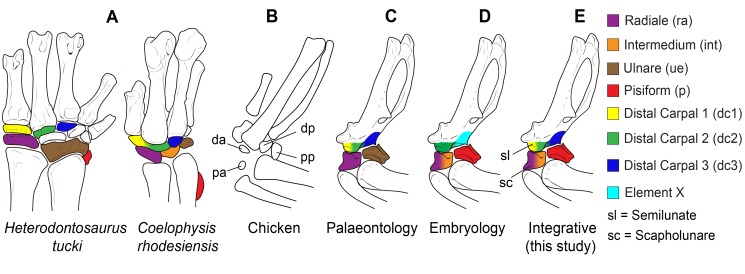
Current hypotheses on the ossifications present in the wrist of birds. (A) The carpal skeleton of early dinosaurs (*Heterodontosaurus, Coelophysis*). Colored elements represent bones that are potentially still present in the avian wrist. (B) The four carpal ossifications of birds as observed in the chicken at 21 d posthatching. The distal–anterior (da) and distal–posterior ossifications thereafter fuse to each other and to the metacarpi. The proximal–posterior (pp) and proximal–anterior (pa) remain unfused. (C) An identification of the four ossifications in the adult chicken wrist as often used in palaeontology. The proximal–posterior ossification is the ulnare (brown), the proximal–anterior ossification is the radiale (purple), the distal–anterior ossification is considered to be a composite of dcI+dcII (yellow+green), and the distal–posterior ossification is considered to be dcIII (dark blue). (D) An identification of the four ossifications in the avian wrist as often used in embryology. The proximo–posterior ossification is the pisiform (red), the proximo–anterior ossification is the radiale+intermedium (purple+orange), the distal–anterior ossification is DCII (green), and the distal–posterior ossification is a neomorphic “element x” (light blue). Despite these general trends, authors in either field may use a different combination of these nomenclatures. (E) Identification of the ossifications in the avian wrist according to the evidence discussed in the present work. We support the use of the term “scapholunare” for the bone that develops from the embryonic cartilage that is composite of radiale+intermedium, and “semilunate” for the ossification that develops from the embryonic cartilage that is a composite of Dc1+Dc2.

The wing of birds is highly derived, having reduced the number of ossifications present in the wrist. Early dinosaurs had as many as nine ossifications (Figure 1A) [1], whereas in birds, only four carpal ossifications remain, two distal and two proximal (Figure 1B) [2]. The two distal ossifications fuse to each other and to the metacarpi in the adult, forming part of the carpometacarpus. The two proximal ossifications do not fuse and are large, independent bones. Currently, the identity of all four ossifications is debatable. Importantly, two classic research fields, palaeontology and developmental biology, often label these bones differently. Figure 1C shows an identification of avian carpal ossifications commonly used in palaeontology, and Figure 1D shows another common for developmental biology, but different combinations of these labels may be found in any field, reflecting current confusion [3]. An important debate also exists over the identity of the digits of the bird wing: Traditionally, palaeontology labels them 1, 2, 3 [4],[5], whereas developmental biology labels them 2, 3, 4 [6]–[11]. In view of recent developmental evidence for 1, 2, 3 [12]–[14], we will use 1, 2, 3 to refer to the digits and, especially so, their associated distal carpals (here, dc1, dc2, and dc3). However, it must be kept in mind that most developmental studies traditionally refer to the same distal carpals as dc2, dc3, and dc4 [3],[8],[15].

**Figure 2:**
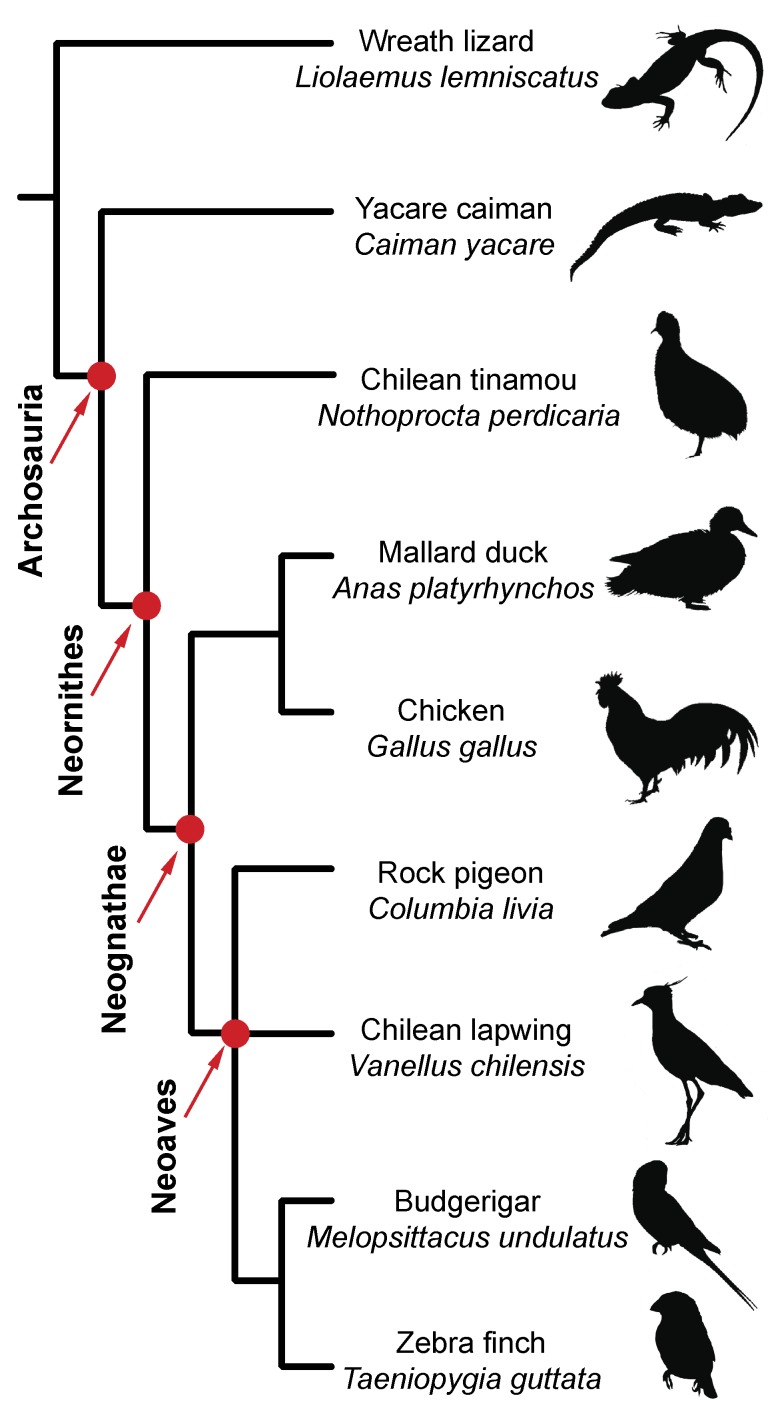
Phylogenetic relationships among the modern taxa used in this study.

Developmental and paleontological data are routinely used for identifying homologies. They often illuminate and support each other, as shown by classic examples such as the bones of the mammalian middle ear [16],[17]. Potential conflicts of data are thus important, demanding for an explanation and coherent integration. Here, we have studied the development of the embryonic wrist skeleton using classic clearing and staining techniques [18] for a broad taxonomic sample of species: wreath lizard, yacare caiman, Chilean tinamou, chicken, mallard duck, rock pigeon, Chilean lapwing, zebra finch, and budgerigar (Phylogenetic relationships among these taxa [19]–[22] are presented in Figure 2). We also used stacks of histological sections to assess tissue organization, such as the presence of an internal separation or “septum” within allegedly composite cartilages. Importantly, we used a new technique for whole-mount immunostaining of proteins expressed within embryonic cartilages. Traditional protocols only allowed antibodies to penetrate cartilage in thin histological sections. We observed the expression in chicken embryos of collagen type II (Coll II), which marks cartilage formation [23]–[26] and collagen type IX (Coll IX), which is indicative of endochondral cartilage maturation [24]–[27]. We also reviewed the paleontological evidence on the carpal bones present during the evolution of the bird line. This included direct observation of specimens in museum collections, especially “bird-like dinosaurs” (the closest nonavian relatives of birds—that is, maniraptorans like Oviraptorosauria, Dromaeosauridae). The integration of our new developmental data with the information provided by the fossil record has important consequences for understanding the evolution of avian wrist bones, leading us to propose a new nomenclature.

## Results

### The Proximal–Anterior Ossification Develops From an Embryonic Cartilage That Is a Composite of Radiale+Intermedium

**Figure 3:**
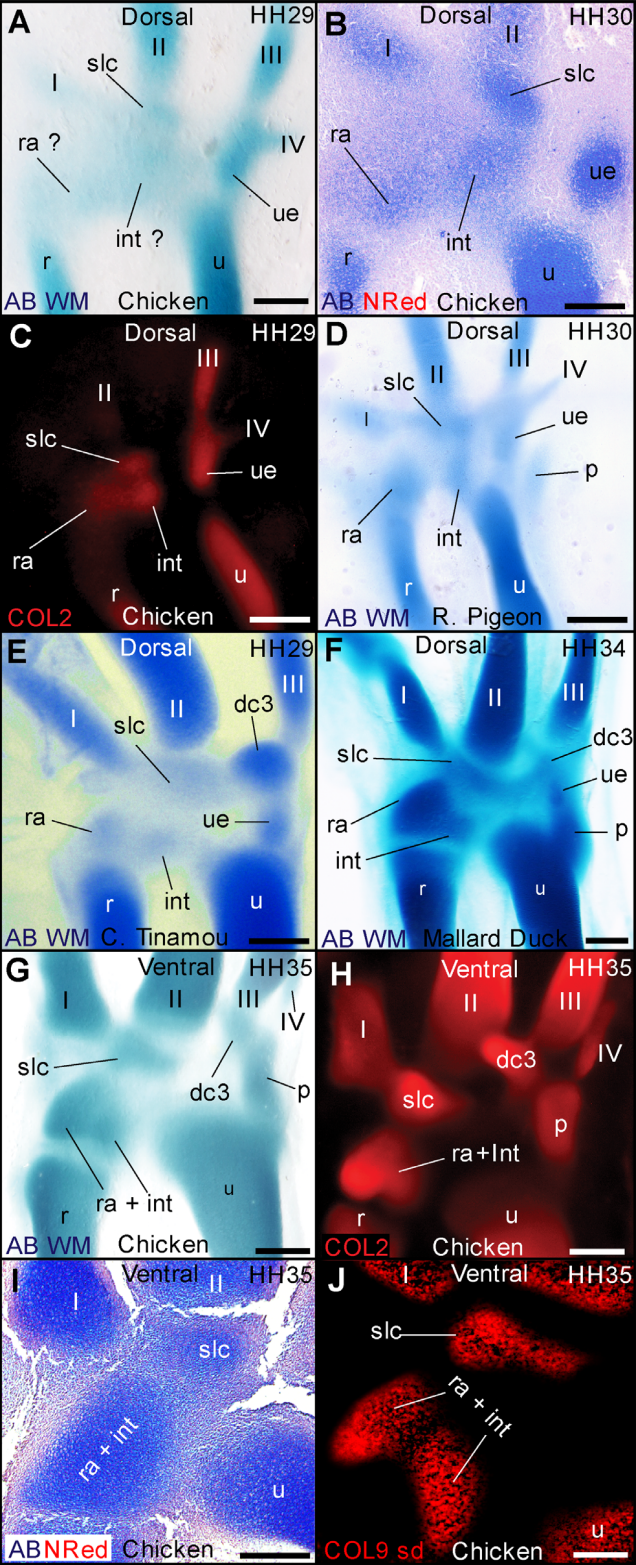
Evidence for a composite radiale+intermedium cartilage in avian embryos. (A) Alcian blue in the chicken shows diffuse staining along the anterior-mid region of the proximal carpus, providing no evidence for a separate radiale and intermedium. (B) Histological sections in the chicken, however, reveal two distinct cartilaginous foci. (C) Immunofluorescence for collagen type II also reveals two separate foci of early expression. (D) Alcian blue is sufficient to observe a separate radiale and intermedium in the development of the pigeon and (E) Chilean tinamou. At later stages, the bilobed shape of the proximal–anterior cartilage suggests it contains the radiale and intermedium in the duck (F) and in (G) the chicken. (I) Collagen type II immunoflourescence also reveals a bi-lobed shape. (H) A histological section of a late stage in the chicken reveals a single perichondrium, with no internal division or septum. (J) Despite this, two separate domains of collagen type IX expression are very distinct, as observed using spinning disc microscopy. These results confirm the composite nature (radiale+intermedium) of the cartilage that gives rise to the proximal–anterior ossification. Scale bars, (A, B, and F) 300 µm, (C) 400 µm, (D–I) 500 µm, and (J) 200 µm.

Developmental studies are unclear on the identity of the proximal–anterior carpal bone (anterior = medial). Some describe it as developing from a single radiale cartilage [6],[8], whereas others describe a composite of the radiale+intermedium cartilages [15],[28]. In palaeontology, this bone is often labelled as the radiale in birds and bird-like dinosaurs, whereas ornithologists often use the term “scapholunare,” a composite of the mammalian terms scaphoid (radiale) and lunare (intermedium) [29]. Whole-mount alcian blue staining in the chicken and budgerigar provides no evidence for two distinct elements, although diffuse staining is present in the entire anterior–central region (Figure 3A), where both radiale and intermedium would be in other amniotes [28]. However, tissue organization in histological sections of chicken reveals two separate elements (Figure 3B). Whole-mount immunofluorescence also reveals two distinct regions of Coll II expression at early stages (Figure 3C). Traditional techniques for visualizing cartilage stain hyaluronic acid and glycosaminoglycans, which are highly concentrated in cartilage but are also present in other connective tissues [30]. Alcian blue often leads to diffuse staining and uncertainty on the number and limits of elements present. Coll II, in contrast, has only been reported in cartilage [23],[26], which may explain why we sometimes found more specific foci within larger domains of weak or diffuse alcian blue staining. Importantly, we found that in early embryos of duck, pigeon, tinamou, and zebra finch, whole-mount alcian blue staining is sufficient to observe a separate radiale and intermedium (see pigeon and Chilean tinamou in Figure 3D,E). This condition was also previously reported in falcons, but no data were shown [28]. Thus, evolutionary variation is present, with greater coalescence of these cartilages in the chicken (Galliformes) and budgerigar (Psittaciformes). These findings illustrate the advantages of observing several species: Some patterns of skeletogenesis are more easily detected in nonmodel taxa. At later stages, a single cartilage is apparent. However, the shape of this cartilage presents two distinct “lobes” (Figure 3F,G) that are also observable using Coll II expression (Figure 3H). Histological sections in chicken indicate complete coalescence, with no septum or traces of separation, and a continuous cartilage matrix surrounded by a single perichondrium (Figure 3I). However, two very distinct regions of late Coll IX expression are present within this cartilage. We confirmed these are largely separate domains using spinning-disc microscopy and 3D reconstruction, avoiding the effects of shape and superposition (Figure 3J). Coll II is expressed upon cartilage formation, but Coll IX relates to cartilage differentiation: after cartilage formation, but before hypertrophy [24],[31]–[33]. Accordingly, we have observed that the onset of Coll IX expression occurs after that of Coll II, and never outside boundaries of larger Coll II expression domains.

### The Distal–Anterior Ossification Develops From an Embryonic Cartilage with Two Distinct Domains of Collagen Expression

**Figure 4:**
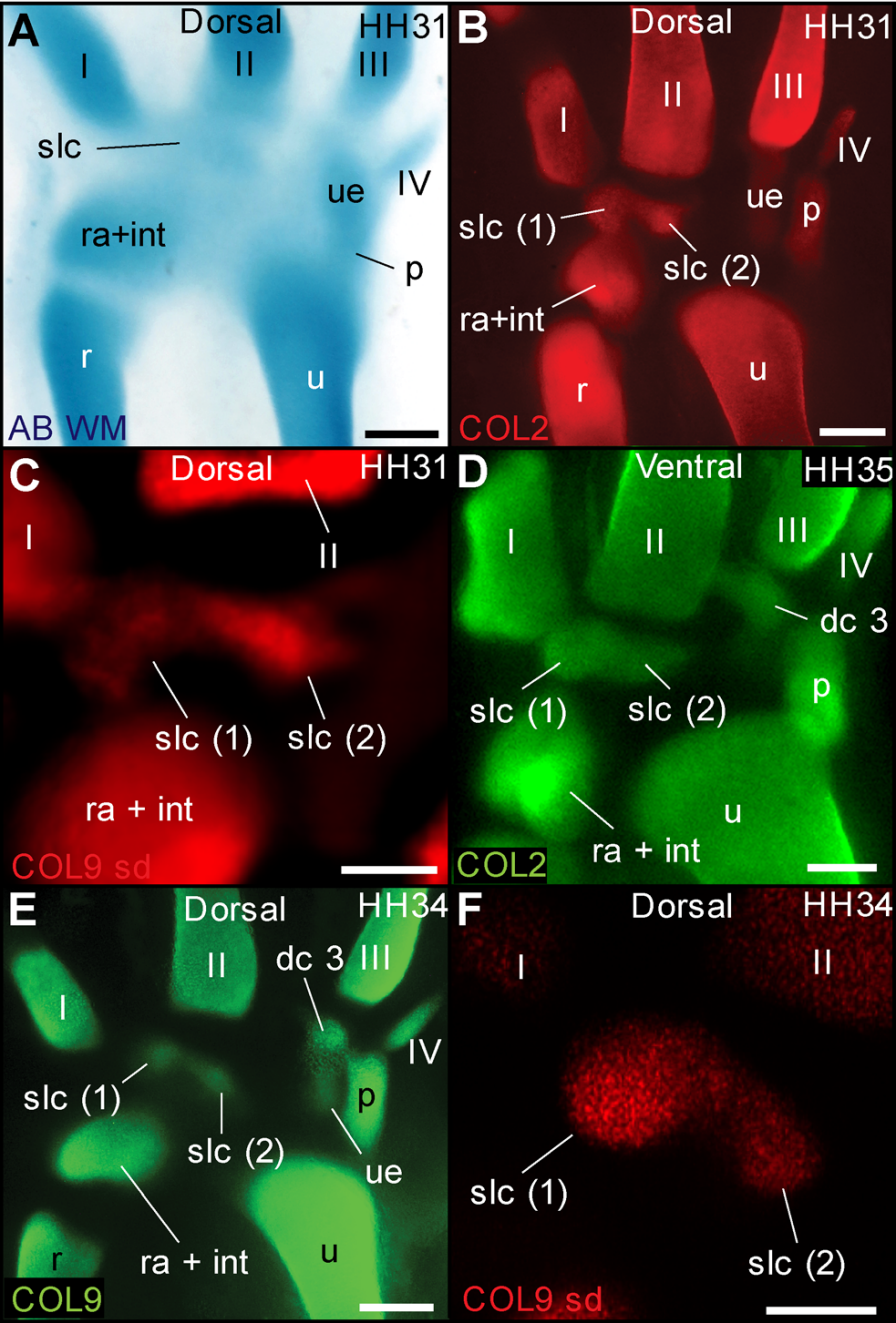
Two regions of collagen expression support the composite nature of the cartilage that becomes the distal–anterior ossification. (A) Whole-mount alcian blue staining in the chicken and all species observed provides no evidence for separate cartilages in the diffusely stained region where the distal–anterior ossification will form (labelled slc; see also Figure 4A–B). However, (B) collagen type II and (C) collagen type IX in this region show two distinct regions of early expression. (D) Later, collagen type II expression becomes more continuous (see also Figure 4C), but collagen type IX expression (E) reveals two nearly separate regions, shown in detail in (F) using spin disc microscopy (see Video S1). Scale bars, (A) 300 µm, (B and D) 400 µm, (C) 200 µm, (E) 500 µm, and (F) 100 µm.

**Figure 5:**
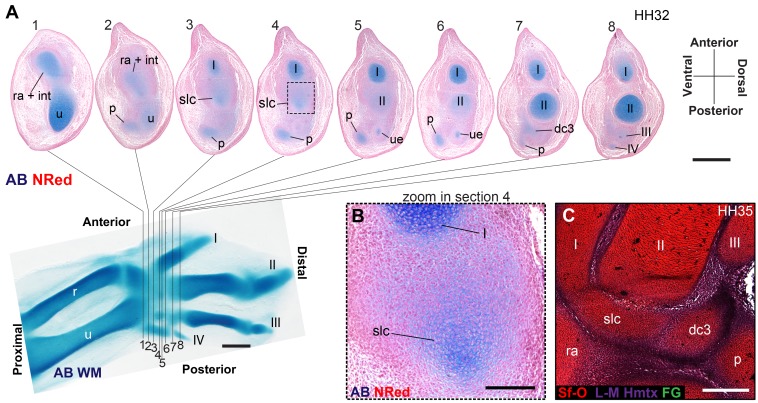
Traditional techniques for cartilage visualization in the region giving rise to the distal–anterior ossification. (A) Stacks of anterior–posterior histological sections, with zoom-in to one section (B) revealing asymmetric tissue organization, with a concentric focus of cells and stronger alcian staining towards posterior. (C) A section in a dorso-ventral stack of a later stage reveals a well-defined cartilage (stained with safranin red) with a single perichondrium and no internal septum or separation. Scale bars, (A and B) 500 µm and (C) 1 mm.

Developmental studies have identified a single distal carpal 2 (dc2) cartilage, at the proximal end of metacarpal 2 [6]–[8], which gives rise to the distal–anterior ossification of birds. Palaeontologists label this ossification as the semilunate, a bone that in dinosaurs is a composite of dc1+dc2. In embryos of the multiple bird species we observed, traditional whole-mount alcian blue staining shows a single region of continuous staining, providing no evidence for a composite of two elements (Figure 4A). Histological sections at early stages of the chicken are ambiguous, revealing asymmetric tissue organization in this region, with weak alcian blue staining towards anterior and strongly stained, concentrically arranged cells towards posterior (posterior = lateral) (Figure 5A–B). However, both Coll II and Coll IX in the chicken show two distinct regions of expression (Figure 4B and 4C). At later stages, uniform Coll II expression indicates the cartilage matrix is continuous (Figure 4D), and histological sections show a single, well-defined cartilage with no internal separation (Figure 5C). However, Coll IX expression continues to show two very distinct, mostly separate regions (Figure 4E), as confirmed by 3D spinning disc microscopy (Figure 4F and Video S1).

### The Embryonic “Element x” That Gives Rise to the Distal–Posterior Ossification Is a Distal Carpal 3, Not a Neomorph Replacing the Ulnare

**Figure 6:**
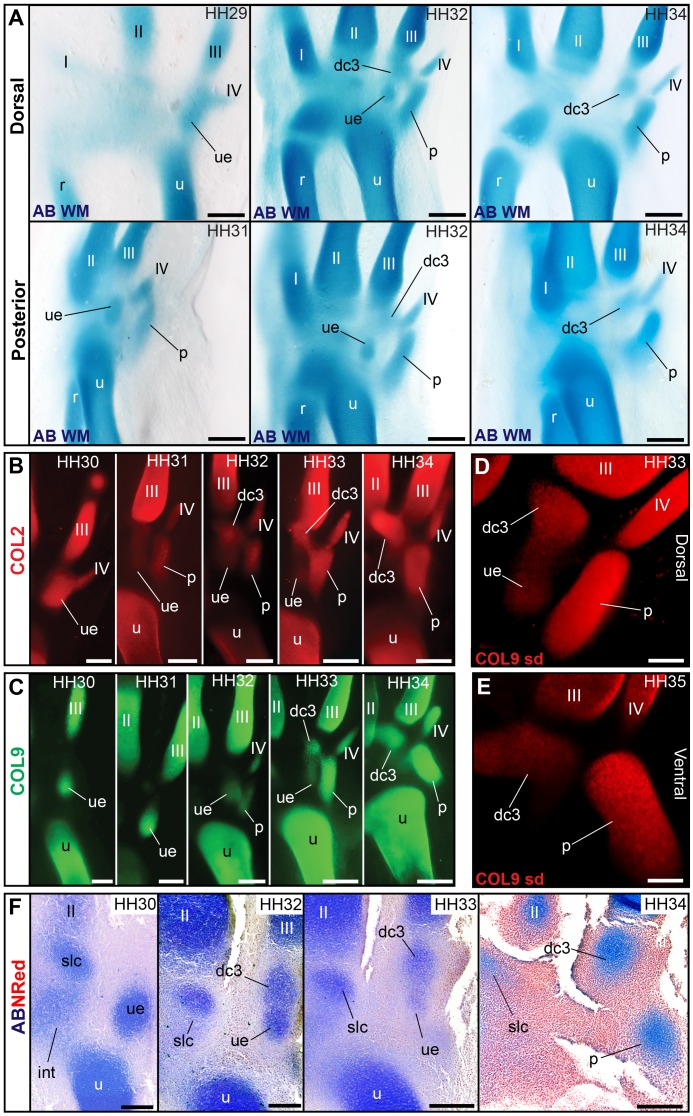
Loss of the ulnare and late formation of distal carpal 3 (“element x”) in the chicken. (A) Whole-mount alcian blue staining confirms the ulnare is the first carpal formed in avian embryos, distal to the ulna. Thereafter, a distal carpal 3 (referred to as “element x” in previous embryological descriptions) is formed distal to the ulnare, coexisting with it. Finally, the ulnare disappears, whereas dc3 persists. (B) Collagen type II and (C) collagen type IX whole-mount immunostaining documents the formation of dc3 distal to the ulnare and the reduction and disappearance of the ulnare. (D) Detail of dc3 and receding ulnare, coexisting in the chicken embryo, as observed by spin-disc microscopy. See Video S2. (E) Detail of dc3 after disappearance of the ulnare. The dc3 cartilage thereafter acquires a bent, “v”-like shape in galloanserae (chicken and duck), but not other bird species observed (Video S3). (F) Histological sections showing the late formation of dc3, its co-existence with the receding ulnare, and the disappearance of the ulnare in the chicken embryo. Scale bars, (A–C and F) 300 µm and (D and E) 150 µm.

**Figure 7:**
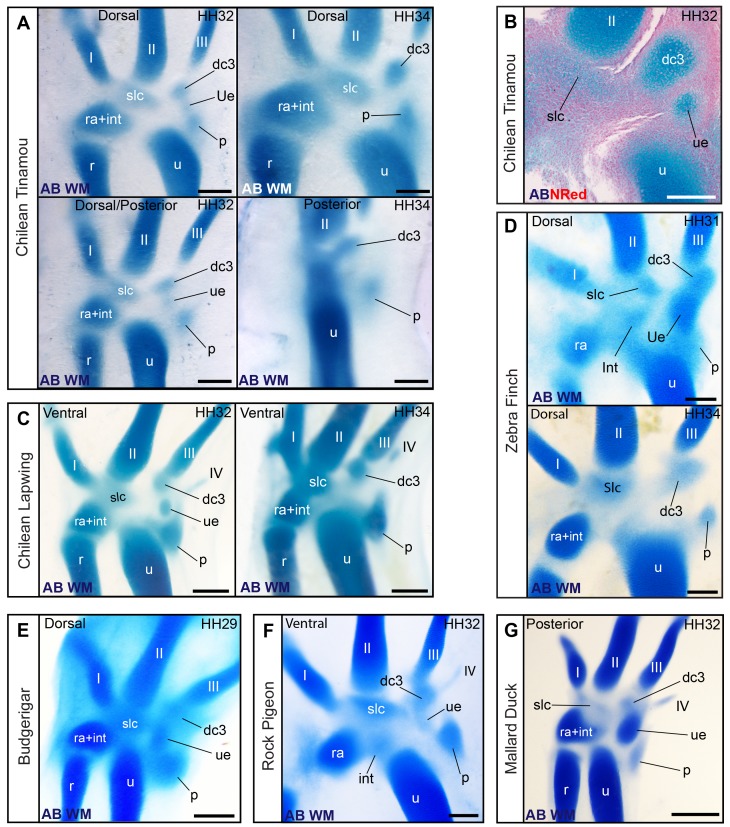
Coexistence of dc3 and the ulnare in a diverse sample of avian taxa. (A) Whole-mount alcian blue staining in the Chilean tinamou showing co-existence and subsequent disappearance of the ulnare. (B) Histological section in a dorso-ventral stack of the Chilean tinamou showing coexistence of the ulnare and dc3. (C) Whole-mount alcian blue staining showing coexistence of the ulnare and dc3 in the Chilean lapwing. (D) Coexistence of ulnare and dc3 and disappearance of the ulnare in zebra finch. (E) Coexistence of ulnare and dc3 in (E) budgerigar, (F) pigeon, and (G) duck. Scale bars, (A and C) 400 µm, (B) 200 µm, (C, G, and F) 500 µm, and (E–D) 300 µm.

In birds, the embryonic cartilage of the ulnare forms early as is typical for amniotes, being the first carpal element formed, at the distal end of the ulna (Figure 6A). We confirm previous reports [8],[34] that the ulnare thereafter ceases to grow and is lost in development (Figure 6A–D). In the chicken, a late-forming cartilage has been described to “replace” the ulnare, which has been called “element x,” suggesting it is a neomorphic element of birds [6]. This cartilage gives rise to the distal–posterior ossification in the bird wrist. These descriptions did not document whether “element x” is formed after the disappearance of the ulnare. We now present evidence that “element x” temporally coexists with the ulnare in the chicken, as observed using alcian blue whole mounts (Figure 6A; “element x” is labelled as Dc3), collagen expression (Figure 6B–C and Video S2), and histological sections (Figure 6D). “Element x” and its coexistence with the ulnare was also observed in alcian blue whole mounts of tinamou, lapwing, pigeon, budgerigar, zebra finch, and duck (Figure 7). Although “element x” has been argued to “replace” the ulnare, we find this notion is misleading, as in all species observed it is distal to it and at the proximal end of metacarpal III, a position that corresponds to distal carpal 3. This is especially evident in the Chilean lapwing (Figure 7C, HH32). Embryonic cartilages of distal carpals can form late, proximal to the preexisting metacarpi [35], as observed for dc1 of the alligator [36]. Thus, we find no compelling reason to consider “element x” is neomorphic or a replacement of the ulnare. Rather, the term “distal carpal 3” is appropriate for this cartilage and the posterior–distal ossification that thereafter develops from it.

### New Developmental Evidence Confirms the Proximal–Posterior Carpal of the Adult Wing Is a Pisiform

**Figure 8:**
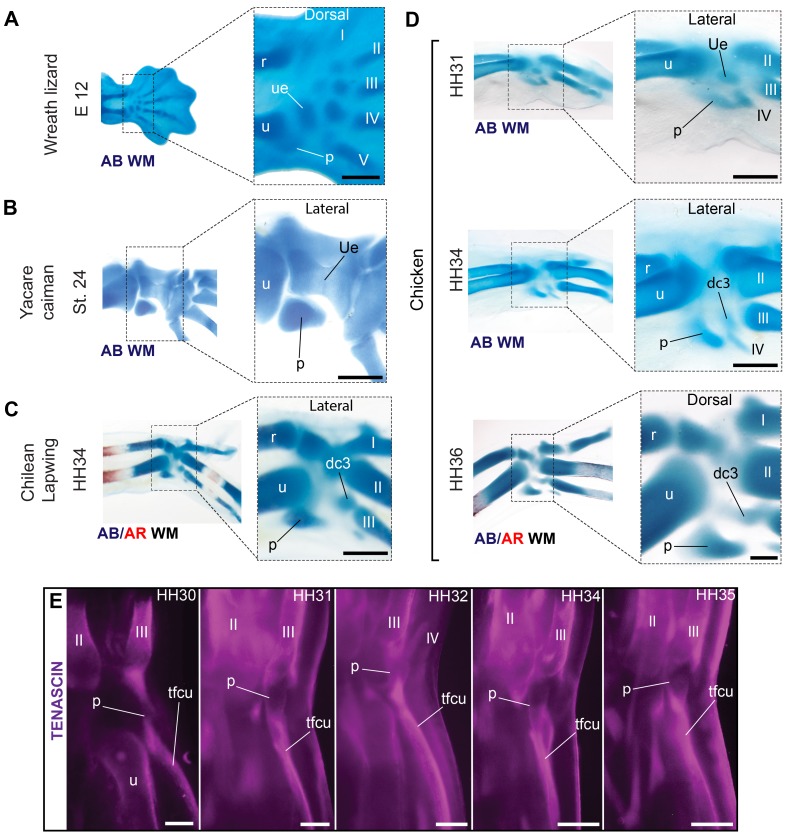
The posterior–proximal ossification of the wing develops from the embryonic cartilage that corresponds to the pisiform of reptiles. (A) The pisiform in embryos of the Wreath lizard and (B) a caiman demonstrates its typical position for amniotes, postero-ventral to the connection of the ulna and ulnare. The cartilage that gives rise to the proximal-posterior bone is found in a comparable position in birds, as shown for (C) Chilean lapwing and (D) a developmental series of chicken. (E) Immunofluorescence for tenascin shows the development of this cartilage is always associated to the tendon of the flexor carpi ulnaris muscle (tfcu), at the turn of the wrist joint, confirming it is a sesamoid, as predicted for the pisiform. Scale bars, (A) 200 µm, (B) 1 mm, (C and D) 500 µm, and (E) 300 µm.

Although the proximal–posterior carpal of birds is often identified as the ulnare in palaeontology, the embryonic ulnare is actually lost during avian development (above section, Figures 6 and 7). Most developmental studies identify the proximal–posterior bone as the pisiform [6],[8]. Our observations confirm it originates from the embryonic cartilage that forms ventrally displaced and posterior to the contact between the ulnare and the ulna, a position that gives rise to the pisiform in other amniotes (Figure 8A–D). The pisiform is a sesamoid that forms associated to a tendon at an articulation joint [37],[38], much like the patella in the knee. In monotremes, marsupials, placentals, turtles, lepidosaurs (tuatara and “lizards”), and crocodylians, this tendon belongs to the *flexor carpi ulnaris* muscle, which begins from the *epicondylus ventralis* of the humerus, glides through the proximal end of the ulna, and attaches to the posterior side of the pisiform [39]–[47]. Immunosflourescence for tenascin confirms that the corresponding embryonic muscle of birds is attached posteriorly to the cartilaginous precursor of the proximal–posterior bone during its formation (Figure 8E), indicating it is a sesamoid, as expected for a pisiform.

### Integration of Paleontological Data

**Figure 9:**
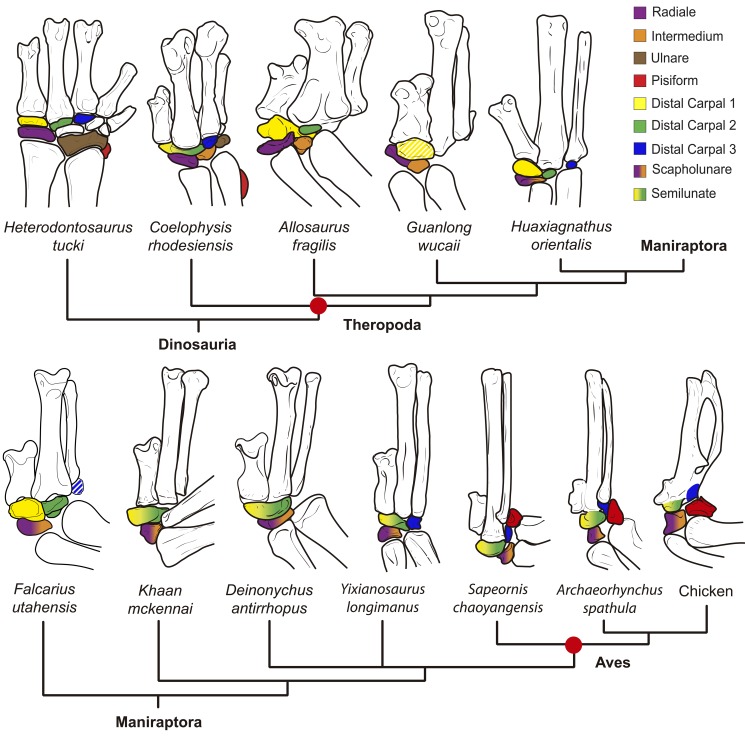
The evolution of the wrist bones in the lineage leading to birds. Incomplete coloring (striped) indicates uncertain identification. A separate ossification of the intermedium (orange) is rarely observed in dinosaurs, but when present, it is seen closely appressed or fused to the radiale (purple). In Maniraptora, a single ossification is present that is commonly referred to as the radiale. However, in birds it develops from a composite radiale+intermedium cartilage and is referred to as the scapholunare. Thus, we propose the use of the term scapholunare for this ossification in bird-like dinosaurs (purple–orange). The distal-anterior ossification of birds (yellow-green) is homologous to the composite semilunate of dinosaurs. In early dinosaurs and most basal theropods, distal carpal 1 (yellow) and 2 (green) were separate bones. The semilunate bone of maniraptoran dinosaurs such as *Deinonychus antirrhopus* covered the proximal ends of metacarpal 1 and 2, and is thus considered to be a composite of dc1+dc2. This is consistent with our new developmental evidence that this bone in modern birds develops from a composite cartilage (Figure 4). Dc1 of *Guanlong* (uncertain, incomplete yellow) could arguably be a semilunate (dc1+dc2). Birds re-evolved a large, ossified pisiform (red). The pisiform and the ulnare were present in early dinosaurs, but thereafter they are not preserved, suggesting that if not absent, they were very small or failed to ossify, consistently escaping preservation. In birds, developmental evidence conclusively demonstrates that the ulnare is lost, but the pisiform is present. A large pisiform is frequently preserved in articulated fossil specimens of birds. The distal–posterior ossification (blue) fuses to the carpometacarpus during the late ontogeny of modern birds. Despite claims it is a neomorphous replacement of the ulnare, its position and development corresponds to dc3, which is found as an independent bone in early dinosaurs, several theropods, and Mesozoic birds (dc3 in *Falcarius* has also been suggested to be an intermedium).

The evolution of the wrist bones in the lineage leading to birds since early dinosaurs is summarized by the taxon sample shown in Figure 9, including phylogenetic relationships [48]–[51]. Regarding the identity of the proximal–anterior bone, our data have confirmed it develops from an embryonic cartilage that is a composite Radiale+Intermedium. A separate ossification of the intermedium (orange in Figure 9) has been described in some theropods such as *Coelophysis rhodesiensis*, *Gorgosaurus libratus*, and *Guanlong wucaii* [52]–[55]. Its presence has sometimes been overlooked, as in *Acrocanthosaurus atokensis* and *Allosaurus fragilis*, where it was mistakenly identified as the ulnare [56]–[59]. In all these taxa, the ossification of the intermedium is closely appressed or fused to the posterior aspect of the radiale (purple in Figure 9), providing evidence that is consistent with the evolution of a composite radiale+intermedium in birds (purple–orange in Figure 9).

In the cartilaginous region that becomes the distal–anterior bone of the bird wrist, the presence of two domains of collagen expression is especially significant when paleontological data are integrated. This bone is comparable to the semilunate carpal of bird-like dinosaurs, which covered the proximal ends of both metacarpals I and II, and is considered a composite of dc1+dc2. In early lineages like *Allosaurus fragilis*, dc1 and dc2 were separate ossifications (yellow and green, respectively, in Figure 9). In some coelurosaurs such as *Harpymimus okladnikovi*, *Alxasaurus elesitaiensis*, and *Falcarius utahensis* [60]–[64], it presented a clear midline suture, indicating the presence of two roughly equal, fused ossifications of dc1 and dc2. In taxa closer to birds, and in Mesozoic birds, a suture line is no longer observable, suggesting a single ossification [65], although the suture may have been lost through bone remodelling during ontogeny [66].

The labelling of the ulnare reveals an apparent contradiction between palaeontology and developmental biology. Most paleontological papers identify the ulnare as present in the bird wrist. Previous embryological studies, however, described the embryonic ulnare was lost and “replaced” by a neomorphic “element x” or pseudoulnare. This complex process was not well documented, allowing for skepticism. According to our developmental data, “element x” is actually dc3, which becomes the posterior–distal ossification: Whether it is a replacement of the ulnare is debatable (see above sections, Figures 6 and 7). However, we fully confirm that the embryonic ulnare is lost in avian development. This provides a strong reason to reexamine the evidence in a broad set of fossil taxa for labelling this bone as being present in birds. Indeed, except in the earliest lineages of theropod dinosaurs [67]–[69] and possibly the Ornithomimosauria [63],[70], there is no evidence of an ulnare (brown in Figure 9). Importantly, there is no ulnare in the most bird-like dinosaurs (Oviraptorosauria, Dromaeosauridae, Troodontidae [71]–[75]), which are known from several well-preserved, articulated specimens (Figure 9). In many theropods, the ulnare was mistakenly considered present, having been confused with other elements, such as the intermedium [56], distal carpal 2 [76]–[78], and the posterior–distal dc3, which in modern adult birds fuses to the carpometacarpus [79],[80]. In early dinosaurs, some bird-like dinosaurs, and Mesozoic birds, dc3 is observable as a separate bone (blue in Figure 9) that has been variably labelled as the ulnare, “element x” [81]–[84], or dc3 [85].

The proximal–posterior bone of the bird wrist (red in Figure 9) poses the greatest challenge to interdisciplinary integration. Paleontological data would seemingly exclude the hypothesis that it is a pisiform, because it provides evidence for its loss in the lineage leading to birds. Except for early theropods [52], and possibly the Ornithomimosauria [63],[86], the pisiform is absent. The most bird-like dinosaurs show the presence only of the semilunate, the scapholunare (often labelled “radiale”), and occasional preservation of dc3 [87], but no pisiform (Figure 9). Thus, if present, the pisiform must have been at least very small or nonossified, consistently escaping preservation. Developmental data, in turn, provide compelling evidence that the large posterior–proximal ossification of modern birds (often preserved in their fossil relatives) is in fact a pisiform in terms of its embryological position, its sesamoid nature, and its muscular connectivity. An integrative explanation for both developmental and paleontological evidence is that a large, ossified pisiform was reacquired in the evolution of birds, after a period in which it was at least strongly reduced (Figure 9). Its evolutionary reappearance as a large, posteriorly displaced proximal carpal occurred early in the evolution of birds, consistently observed in Mesozoic taxa such as the cretaceous long-tailed bird *Shenzhouraptor sinensis*, the basal pygostylians *Sapeornis chaoyangensis* [83], and *Confuciusornis sanctus* ([88], personal observation). A bone in appropriate position has been reported in the Eichstätt specimen of *Archaeopteryx* [89], but not other specimens [90]. In other Mesozoic taxa closer to modern birds (Ornithothoraces, [91]–[94]), this bone became v-shaped, like the pisiform of modern birds [95],[96].

## Discussion

Although the anterior–proximal carpal of modern birds (purple–orange in Figure 9) develops from a composite radiale+intermedium cartilage (Figure 3), a single ossification is formed [2] that cannot be attributed to either the radiale or the intermedium by itself. However, the use of a “radiale+intermedium” label for this bone could misleadingly suggest two fused ossifications, overlooking the evolutionary simplification to a single ossification, in itself an important innovation. Thus, we support the use of a special name for this bone. The available term “scapholunare” may provide an appropriate choice. In bird-like dinosaurs and Mesozoic birds, no separate intermedium has ever been reported, suggesting that reduction to a single ossification had already occurred. In these fossil taxa, this bone is commonly identified as the radiale, but we suggest the term scapholunare may also be used, under the argument that the best inference about its development is provided by their closest living relatives.

The morphological similarity of the anterior–distal carpal bone (semilunate, yellow–green in Figure 9) in Mesozoic birds like *Archaeopteryx* and maniraptoran dinosaurs such as *Velociraptor* is one of several skeletal traits traditionally used to support the descent of birds from dinosaurs [5],[71],[97],[98]. Because previously available developmental data showed one ossification forming from a single dc2 cartilage, this fact was used to argue this element in birds was not homologous to the semilunate of dinosaurs, and thus could not support their relatedness to birds [99]. Within acceptance of the dinosaur–bird link, it is also discussed that the semilunate of bird-like dinosaurs and early birds could represent only one enlarged distal carpal [100],[101]. In this context, the presence of two distinct domains of collagen expression (Figure 4) provides compelling new support for direct homology of this bone to the composite semilunate of dinosaurs. As in the case of the scapholunare, rather than using dc1+dc2, we support labelling the ossification of modern birds with the same special term “semilunate” used for this bone in bird-like dinosaurs and Mesozoic birds.

Developmental data exclude the hypothesis that the posterior–proximal ossification (red in Figure 9) is the ulnare, which disappears (Figure 6), and instead shows it is derived from the embryonic cartilage identified as “element x.” Our reexamination of the developmental evidence provides no support for “element x” being a neomorph that somehow replaces the ulnare (Figure 7). Rather, because the embryonic position of “element x” actually corresponds to dc3, we support labelling this cartilage and its ossification as dc3 (blue in Figure 9), instead of “element x,” in both modern birds and their fossil relatives.

Developmental evidence strongly supports the identification of the proximal–posterior bone of birds as the pisiform. In quadrupedal reptiles, the pisiform is large and important for locomotion [44],[102]. In birds, the pisiform is functionally important for bird flight: It articulates proximally with the ulna, and distally with the carpometacarpus, transmitting force during the wing downstroke, and restricting flexibility during the upstroke [103],[104]. The evolutionary reappearance of a large ossified pisiform in early Avialae (red in Figure 9) suggests its relation with the early evolution of flight and the reinvolvement of the forelimb in locomotion. Although it can be argued that the proximal-posterior carpal of birds should be considered a neomorphic bone, this description hides the fact that its muscular connectivity and embryological origin are identical to the pisiform of other reptiles. Thus, we support labelling this bone in birds as the pisiform.

### Conclusion

The development of living species is expected to contain signs of their evolutionary lineage of origin. Because radically different data sources about evolution are available (fossils vs. molecular and cell biology), transdisciplinary integration provides a great opportunity for independent confirmation. Several examples exist where molecular-developmental observations show great consistency with the information provided by the fossil record [105]–[107]. Sometimes, however, each area seemingly arrives to a different conclusion. It often occurs that one of the data sources needs revision or updating. However, when all facts are well documented, apparent contradictions may point to the need for a different interpretation. For instance, an explanation may be found in a previously unsuspected evolutionary transformation [12],[108].

In the case of the bird wrist, a renewed look found support in both data sources for a composite radiale+intermedium, which had often been simply labelled as the radiale in both fields. The evidence for a composite semilunate cartilage shows how, despite claims to the contrary, avian development contains signals that are consistent with their origin from dinosaurs, which is a well-documented fact of palaeontology. Our detailed confirmation of the developmental loss of the ulnare led us to reexamine updated evidence from the fossil record. Paleontological evidence in fact strongly supports the loss of the ulnare in the bird line, ultimately revealing no inconsistency with developmental data. Perhaps the most interesting result of combining data sources is provided by the case of the pisiform. Sound fossil evidence indicates this ossification was absent in bird ancestors, but using developmental evidence alone would decidedly identify this bone as present in modern birds. The evolutionary reacquisition of a large ossified pisiform in birds can explain how both data sources could in fact be correct. The notion of important evolutionary reversals has historically met a lot of resistance in evolutionary thinking [109]. Although its empirical reality is now accepted [110], it continues to be considered an oddity [111]. The reappearance of the pisiform in birds provides a compelling case documenting this intriguing evolutionary phenomenon. Integrating developmental and paleontological information can thus also be informative about what evolutionary processes are actually possible. These transformations would be hard to detect using only one source of information.

Palaeontology and developmental biology often have radically different research objectives and methods. However, they intersect significantly. The avian wrist provides a striking new example of how they can illuminate each other in concrete ways. This is reflected in our updated proposal on the identity of bird wrist bones (Figure 1E). Evolution, as documented by the fossil record, provides natural experiments that are outputs of the same developmental mechanisms that are conserved in living organisms. A complete separation of development and palaeontology misses opportunities for understanding evolution, much like a separation of astronomy and experimental physics would delay the advances of cosmology.

## Materials and Methods

### Experimental Animals and Museum Collections

All procedures were formally approved by the Comité de Etica de la Facultad de Ciencias, Universidad de Chile, which certifies compliance with all aspects required for government funding (http://www.conicyt.cl/fondecyt/2012/10/31/bioetica/). Live animals were only kept to obtain fertilized eggs: None were euthanized or used for experiments. None of the wild species used is in a conservation category of concern (http://www.iucnredlist.org), and eggs were taken with field permits of the Servicio Agrícola y Ganadero (SAG, Government of Chile). Fertilized eggs of *Gallus gallus* (Chicken, Galliformes), *Anas platyrhynchos* (Mallard duck, Anseriformes), and *Nothoprocta perdicaria* (Chilean tinamou, Tinamiformes) were purchased from local farms: Chorombo S.A, Avícola Metrenco, and Tinamou Chile (perdiz.cl). Fertilized eggs of *Columba livia* (Rock pigeon, Columbiformes), *Taeniopygia guttata* (Zebra finch, Passeriformes), and *Melopsittacus undulatus* (Budgerigar, Psittaciformes) were obtained from birds kept at facilities of the Faculty of Science, University of Chile. Eggs of *Liolaemus lemniscatus* (Wreath lizard, Iguania) were obtained from gravid females captured and then liberated with SAG permit (AOV), and were incubated with the same protocol for *Liolaemus tenuis* [112]. *Caiman yacare* embryos (Yacare caiman, Alligatoridae) belong to Paula Bona (Museo de La Plata, Argentina). Fertilized eggs of *Vanellus chilensis* (Chilean lapwing, Charadriiformes) were collected with permission from SAG (D.S.-P. and D.N.-L.).

The following specimens and casts of fossil taxa were observed directly: American Museum of Natural History, New York: *Archaeopteryx lithographica* (Avialae) cast FR 5120 (Berlin specimen) and cast FR 9495 (Eichstatt specimen), *Bambiraptor feinbergi* (Dromaeosauridae) AMNH 30554, *Citipati osmolskae* (Oviraptorosauria) TG002 IGM 100/978+3063, *Coelophysis bauri* (Coelophysoidea) 30631, and *Khaan mckennai* (Oviraptorosauria) MPC-D 100/1002, MPC-D 100/1127; Natural History Museum of Los Angeles County: *Confuciusornis sanctus* (Confuciusornithidae) cast LACM 7852; Museo de Ciencias Naturales, Universidad Nacional de San Juan: *Herrerasaurus ischigualastensis* (Herrerasauridae) PVSJ 373; University of Texas: *Coelophysis bauri* (Coelophysoidea) MCZ 4329; University of California Museum of Paleontology, Berkeley: *Dilophosaurus wetherelli* (Coelophysoidea) UCMP 37302; and Peabody Museum of Natural History, Yale University: *Deinonychus antirrhopus* (Dromaeosauridae) YPM 5206, *Tanycolagreus topwilsoni* (Coelurosauria) cast YPM 56523.

### Cartilage Staining

Embryos were fixed in 100% methanol for 2–3 d at room temperature (RT). Methanol was replaced by 5∶1 ethanol/acetic acid solution with 0.03% 8G alcian blue for 2 d at RT in an orbital shaker. Then, embryos were cleared in a sequence of 1∶3, 1∶1, and 3∶1 glicerol/water, and photographed in a stereoscopic microscope. Two embryos per stage were used for Chilean lapwing. Five embryos per stage were used for Chilean tinamou, duck, and pigeon. Two or more embryos per stage were used for zebrafinch, budgerigar, and chicken. Three embryos per stage were used for Wreath lizard, and one for Caiman.

### Serial Histological Sections

Embryos were fixed in 4% paraformaldehyde (PFA) for 2 h at RT or overnight at 4°C. Then, forelimbs were dissected, washed in PBS 1%, and dehydrated in ethanol increasing concentrations (50% to absolute ethanol). Limbs were cleared in Neoclear and vacuum-embedded in Paraplast for 1 h. The paraplast-embedded material was cut into 10–12-µm-thick sections, rehydrated, and stained with alcian blue/nuclear red or safranin/hematoxylin.

### Whole-Mount Immunofluorescence

Six embryos for each stage were used for immunofluorescence of each primary antibody. Embryos were fixed in Dent's Fix (4∶1 methanol/DMSO) for 2 h at RT, dehydrated in 100% methanol, and left at −80°C overnight. Before immunostaining, they were bleached in Dent's Bleaching (4∶1∶1 methanol/DMSO/H2O2) for 24 h at RT. For anticollagen immunostaining, embryos were fixed and bleached as above. Then, limbs were dissected and digested with 2 mg/ml of hyaluronidase (Sigma) in PBS for 2 h at 37°C. Embryos were rehydrated in PBS 1% triton (PBST) and incubated in primary antibodies for 2 d at 4°C in an orbital shaker. Primary antibodies were diluted in 2% horse serum, 5% DMSO in PBST at the following concentrations—1∶10 anti-tenascin (M1-B4, DSHB); 1∶40 Colagen type II (II-II6B3, DSHB); and 1∶40 collagen type IX (2C2-II, DSHB)—and washed in PBST (3×10 min and 3×1 h in an orbital shaker). Secondary antibodies antimouse (Alexa-488 and Alexa-Fluor 594, Jackson ImmunoResearch, PA) were diluted in 5% goat serum, 5% DMSO in PBST and incubated for 24 h at 4°C. After that, they were washed, cleared with Urea 4M, and photographed in a fluorescent stereoscopic microscope (Nikon). For 3D reconstructions, 10 µm stacks were obtained in a spinning disk confocal microscope (Olympus) and projected in cellSens software (Cellsens analysis Z stack was obtained for ·D reconstruction and 2D deconvolution/Nearest Neighbor analysis was obtained for removal of background fluorescence).

## Supporting Information

Video S1 The cartilage that gives rise to the distal–anterior ossification of birds presents two distinct domains of collagen IX expression in the chicken embryo, as observed at stage HH34 of the chicken using a spinning disc confocal microscope.
(MP4)

Video S2 Coexistence of the receding ulnare and the newly formed dc3 in the chicken embryo, as observed at HH32 using a spinning disc confocal microscope.
(MP4)

Video S3 After disappearance of the ulnare, dc3 acquires a bent, “v”-like shape in Galloanserae, as observed at stage HH35 of the chicken using a spinning disc confocal microscope.
(MP4)

## Abbreviations

coll II: collagen type II
coll IX: collagen type IX
dc: distal carpal
int: intermedium
pi: pisiform
re: radiale
sc: scapholunare
sl: semilunate
